# Supplementary material for: Exploring the burden on family caregivers in providing care for their mentally ill relatives in the Upper East Region of Ghana
Source: PLOS Glob Public Health. 2024 Apr 16;4(4):e0003075. doi: 10.1371/journal.pgph.0003075 (PMC11020355; doi:10.1371/journal.pgph.0003075)
Supplement: S1 Text — (DOCX) [file pgph.0003075.s001.docx]

**CODE: 001**

1. Can you please tell me your age? 45

2. What gender are you? female

3. What is your ethnicity? Kasena

4. What is your marital status? married

5. How many children do you have? 2

6. Are you educated? To What level please? Masters

7. What work do you do? teacher

8. What is your place of residence? Navrongo

9. What is your religion? Christian

10. How are you related to the patient? Parent

I: a) Before your relative got mentally ill, what was your thinking regarding mental illness?

R: Well, I have always thought that it is a result of drug abuse, marijuana and other things like that.

I: b) What about your relative made you think he/she was mentally ill?

R: He has always been fine without any issues until I was called one evening by the school that he had been sent to the War Memorial Hospital when I go there he is talking out context and shouting on top his voice and destroying things in the ward. That was when the Drs told be he was having a mental illness.

I: How do people in your community think about mental illness?

R: hmmm, here the stigma is too much, there have been times when people sarcastically ask me , how is your boy doing only to look aside and laugh at me. My son also tells how some of his mates call him the crazy guy.

I: d) What do you think are the causes of your relative’s mental illness?

R: I just don’t know where this came from because all the places, I have been to with him they tell me it’s a mental disorder but are not able to tell me what caused it.

I: a) Where did you visit first for the diagnosis? Why?

R: Well, the school took him to the hospital the first time and that was where we were told he has a mental illness.

I: b) Where did you visit first for treatment? Why?

R: it was the same hospital where he was diagnosed that the treatment was initiated and further referred to Bolgatanga for specialist care. I have only sort medical treatment for him since I believe it is a medical condition,

I: c) What factors discourage you from bringing your relative to the hospital for mental health service

R: There is nothing that discourages me except that this problem is financial draining and very expensive otherwise, there is no way I will not seek care for my son.

I: d) What factors encouraged you to seek mental health care from Hospital?

R: The staff at the Psychiatric hospital are very accommodating even though at another facility they were very hostile and shouting at my son. But at the current place, they treat my son well and so I like going there.

I: e) What benefits have you derived from bringing your relative to the hospital for mental health services?

R: Even though he is not completely well yet, but I know he is better now because of the treatment he has received so far. I think he could have been worst.

I: a) How has the mental illness of your relative affected you? Please explain

R: Oooh, I have suffered a lot, emotionally and financially. It has cost me so much with very expensive investigations. I remember I spent 6000 cedis the last time travelling to Tamale and Accra for investigations. As for the emotional challenges, they are just too much. I find it difficult to concentrate and mostly feeling sad.

I: b) How do you cope with these challenges or stresses you experience? Please explain

R: I don’t have a choice than to encourage myself and move on hoping that things will get better soon otherwise there is nothing more to do. Another thing is prayers.

I: 1. What do you think should be done to address those factors hindering the utilization of mental health services in this municipality?

R: I think the nursing staff should be given a little more training to be well abreast with these conditions. Brain conditions are very stressful hence mistakes need to be avoided.

**CODE: 002**

1. Can you please tell me your age? 68

2. What gender are you? female

3. What is your ethnicity? talensi

4. What is your marital status? married

5. How many children do you have? 4

6. Are you educated? To What level, please? tertiary

7. What work do you do? retirement

8. What is your place of residence? SSNIT

9. What is your religion? CHRISTIAN

10. How are you related to the patient? SON

I: Before your relative got mentally ill, what was your thinking regarding mental illness?

R: Hahahahaa, hmmmm, well, initially I associated it with maybe people who have taken drugs and developed it and along the line through education I also realized that there could be problems during labor that can also lead to other things and that apart, I also heard people also say it could be a curse or maybe a spell that somebody might have cast upon them.

I: What about your relative made you think he/she was mentally ill?

R: His case was, it took a long time for us to detect it was a mental illness. He has always been the calm type so it was during a time I travelled and was called that he fell sick. Robert will not eat, will not drink and will not talk to anybody. Initially I thought it was a different type of sickness. I realized that it began from them when he isolated himself from people. He was diagnosed there as depression and I asked what could have caused this since I have all the time for him. Our only indicator was when he withdrew from people, we know he is the quiet type but this time it was extreme.

I: How do people in your community think about mental illness?

R: Well. Hmmm, just like I am saying, we all thought these are bad buys who took in some drugs and also maybe one might have stolen somebody’s thing and has been cursed or something.

I: What do you think are the causes of your relative’s mental illness?

R: Hmmm, Robert’s case, I cannot attribute it to any particular cause, during the third trimester, they said I had bile in the blood or so but I never missed ANC, my labor delayed for 3 days and when I delivered him, he has a blood stain on the eye is the cornea or so… he had a delay in every aspect, delayed in walking, talking and they also said he had a tongue tie which they even treated. He didn’t have any other problem until I travelled and was called that he fell sick and that was the beginning of the whole thing. I don’t know whether it was the labor or something.

I: Where did you visit first for the diagnosis? Why?

R: The hospital, like io said, I wasn’t in town when I was called that he was sick and upon my return, I was told he had depression

I: Where did you visit first for treatment? Why?

R: Like I said, he was diagnosed in the hospital and treated initiated since, I have never sent him anywhere apart from the hospital

I: What factors discourage you from bringing your relative to the hospital for mental health service

R: I have been a unique parent; I didn’t care about stigma or anything. I always go with him to the hospital and have pushed away everything that people will say and take him to the hospital.

I: What factors that encouraged you to seek mental health care from Hospital?

R: it was my desire that he will recover and I know if he recovers, I can benefit from him so my interest is to give him the necessary assistance so that he can get well and I can also get to my normal life.

I: What benefits have you derive from bringing your relative to the hospital for mental health services?

R: Oh yes, why not, despite the long treatment he has taken and I still see symptoms, I think it could have been worse than this if I have not brought him to the health facility. these symptoms are there he never disturbs and we chat, he is able to carry out his own activities and do his own things. his only challenge is that his mind is not back to think about marriage or something but the health facility has been very helpful.

I: How has the mental illness of your relative affected you? Please explain

R: Ooh greatly, like I told you, I am a daring type and don’t give yup. I planned going forward in education but I had to drop to take care of him. When I want to travel to his other siblings, and leave him alone, he ends up travelling from home and because of that I cannot travel. I also have to be putting some few cedis in his account so that he can withdraw some hundred cedis but not more than that.

I: How do you cope with these challenges or stresses you experience? Please explain

R: My coping? Mmm, well you have to cope now that you know the condition. I have to just make up my mind and come what may do all I can do to take care of him. I am not bothered about what people will say. I just pray and tell God to take care of the condition. In pray that God will help me and anytime I pray God answers me.

I: What do you think should be done to address those factors hindering the utilization of mental health services in this municipality?

R: Yh, I will say that the Hospital has been very helpful. My only challenge is the lack of transport for you to visit the patients at home. It becomes comforting when you see a friend from the health center come to visit you. so that you know that somebody else care and not your mum alone.

**CODE: 003**

1. Can you please tell me your age? 40

2. What gender are you? MALE

3. What is your ethnicity? NANKANE

4. What is your marital status? MARRIED

5. How many children do you have? 3

6. Are you educated? To What level, please? YES, TERTIARY

7. What work do you do? RECORDS OFFICER AT THE DISTRICT ASSEMBLY

8. What is your place of residence? NAVRONGO

9. What is your religion? CHRISTIAN

10. How are you related to the patient? HUSBAND

I: Before your relative got mentally ill, what was your thinking regarding mental illness?

R: My view was that mental illness is treatable, when I met my wife, she did not hide it from me, so I said no problem so we can go ahead with the marriage, my general view is that it is treatable.

I: Apart from your wife’s situation, what is you thinking about mental health in general?

R: Apart from that, in Ghana, the sector has been neglected somehow, government doesn’t pay so much attention to the condition. even the district assembly common fund, mental health has a percentage but they rather concentrate on leap and HIV instead of considering mental health, and that is very bad because it is a critical sector

I: What about your relative made you think he/she was mentally ill?

R: You see, what I have observed so far is that I sometimes advise my wife that we should have time for ourselves, she thinks a lot about little problems. I realized that a review of her salary reducing her take-home salary made her to start thinking a lot.

What symptoms do you notice to show that the condition is coming back

R: when it came, she becomes difficult to understand, like leaving her motorbike on the road and start walking home, attempting to remove her clothes, and also forgetfulness. These are the things I have noticed so far

I: How do people in your community think about mental illness?

R: hmmmm, you see we are in a village where the illiteracy rate is high so any little thing, they say the fellow is made so forget about him or her. They don’t consider them as human beings and do not take them seriously. Generally, they don’t consider them as important in society so generally I will say stigmatization. That what I can say about their thinking about mental illness

I: What do you think are the causes of your relative’s mental illness?

R: she thinks a lot especially when there is a little stress

I: Where did you visit first for the diagnosis? Why?

R: Hospital, that was Accra Psychiatric hospital, her family members are elites so they first suggested hospital and that is where she has been seeking treatment since 1999

I: Where did you visit first for treatment? Why?

R: Hospital, just like I mentioned to you earlier

I: What factors discourage you from bringing your relative to the hospital for mental health service

R: The first day we went to the hospital, I was so discouraged because of the way she was behaving but subsequently, I saw her get batter gradually and that was my motivation to maintain bringing her to the psychiatric hospital

I: What factors encouraged you to seek mental health care from Hospital?

R: Yes, the factors that have motivated me is that I thought that If I send her to a psychiatric hospital, she will get better medicine there, they will handle her well and she will be cured and discharged.

I: What benefits have you derived from bringing your relative to the hospital for mental health services?

R: The benefit is that, we are together and I told you that the condition is treatable. I now when the condition comes, I don’t feel scared or shy again. I also understand the condition better and this is a benefit I derived

I: How has the mental illness of your relative affected you? Please explain

R: It has affected me mentally and physically. I grew lean because I was running up and down, I could not sleep well and could not eat. I couldn’t take care of my children and we spent a lot of money traveling up and down for care in Accra and other places. I always think that I have married my wife and this is happening to me, if she is unable to come up, how will I take care of the children

I: How do you cope with these challenges or stresses you experience? Please explain

R: I always say everything is by God so I pray to God that things will be ok. whatever comes, God will sometimes test you to see the outcome. The 2 of us went to alter to pronounce this marriage so it could be that god is testing me to know if the love is there. So, I feel normal because of God. The family also came in and supported here and there.

I: What do you think should be done to address those factors hindering the utilization of mental health services in this municipality?

R: The government should take this mental health serious. The government should take it up. Most of the people who use the service do not wish to become mad but they are found there. The food and drug authorities should be up and doing.

**004**

1. Can you please tell me your age? 38

2. What gender are you? female

3. What is your ethnicity? frafra

4. What is your marital status? widowed

5. How many children do you have? 3

6. Are you educated? To What level, please? nil

7. What work do you do? unemployed

8. What is your place of residence? sirigu

9. What is your religion? christian

10. How are you related to the patient? Mother

1. Before your relative got mentally ill, what was your thinking regarding mental illness?

I always thought that mental illness could be as a result of come spiritual attacks or it could be something in their family or something. Even my son used to report his friends who smoke marijuana to their parents and even tell them if they don’t stop, they will go mad

1. What about your relative made you think he/she was mentally ill?

Clintons’ behavior just changed. He started giving people cheeky answers, ignoring elderly people and taking things that don’t belong to him. He also started complaining that he hears voices asking him to pick other peoples’ things. He was also seen pacing all over the place

1. How do people in your community think about mental illness?

They always think that mental illness comes from people’s families, others also say is it not weed and alcohol that is destroying them? Any time they see a mentally challenged person and also maybe the person has stolen something and has been cursed with a deity.

1. What do you think are the causes of your relative’s mental illness?

.

1. Where did you visit first for the diagnosis? Why?

I first sent him to the church for prayers because I did not think it could be a medical condition but spiritual hence, I sent him to the church

1. Where did you visit first for treatment? Why

The church, like I mentioned earlier for prayers, it was after the symptoms started coming and going that is when io sent him to the hospital

1. What factors discourage you from bringing your relative to the hospital for mental health service

to kept buying the drugs for his treatment but I want getting the needed response hence I sometimes feel like not taking him to the hospital at all.

1. What factors that encouraged you to seek mental health care from Hospital?

I believe that combining the hospital with prayers is what will work, using only one source doesn’t give the results one requires

1. What benefits have you derive from bringing your relative to the hospital for mental health services?

I have some good results as a result of the use of the service. his symptoms have resolved even though it sometimes recur. It is however better now

1. How has the mental illness of your relative affected you? Please explain

Giving birth is a painful experience so I am always worried about my son’s condition. It gives me a lot of worry. It has also cost me so much money but that is not my focus because I really want my son to get well. Another thing that has affected me is that because of his condition, I am unable to get the time to go and work like before.

1. How do you cope with these challenges or stresses you experience? Please explain

I rely on God as the sole healer and so I console myself with my beliefs

1. What do you think should be done to address those factors hindering the utilization of mental health services in this municipality?

I will encourage all the workers in the service to keep on with their good work.

**005**

1. Can you please tell me your age? 20

2. What gender are you? male

3. What is your ethnicity? frafra

4. What is your marital status? single

5. How many children do you have? nil

6. Are you educated? To What level please? SHS

7. What work do you do? Unemployed / apprentice

8. What is your place of residence? Bolga

9. What is your religion? Christian

10. How are you related to the patient? Uncle

1. Before your relative got mentally ill, what was your thinking regarding mental illness?

R: From my own view, if the person is not taking drugs or smoking or the person is cursed, I don’t think it is something normal.

1. What about your relative made you think he/she was mentally ill?

R: You know when it started if I can remember the time, I was staying with him, when it comes, he will just go out a few days, 5 days, unless people outside who knows him, they will bring him. When it comes, he will just go out, he doesn’t want the family members to come near him. When you call him, he will say I am here but when you get there he will move to another place and say I am here.

1. How do people in your community think about mental illness?

R: It’s just, if you see someone mad or something, you assume that he smokes, when you think about a lot of things or you forget yourself it can cause this problem,

1. What do you think are the causes of your relative’s mental illness?

For that one I don’t know whether it is demons or something but when he talking, he says is like they hate him because of the house and want to take the house from him. To me I will say what he has been saying I will take it like that.

1. Where did you visit first for the diagnosis? Why?

R: I don’t know where they sent him, he was brought back normal. Subsequent ones, they take him to the hospital and they gave him medicines but he didn’t take and that caused all this.

b) Where did you visit first for treatment? Why?

1. What factors discourage you from bringing your relative to the hospital for mental health service

R: First when they sent him to the hospital in tamale and they discharged him he is mind was that he wasn’t sick. That if he was sick, they would have kept him there for a long time but because they admitted him for a short time, he says he is not sick so will not come to the hospital. also, financial challenges in paying for the services and purchasing medications which are not always available for free also prevent us from bringing him to the hospital

d) What factors that encouraged you to seek mental health care from Hospital?

R: Errm, this court case that came around, we need a report so I want to bring him to the hospital so that we can get the report to deal with the court case. Also, when I completed school, I wanted to go the the military and he is the only one who can help me so I want him to get well.

1. What benefits have you derive from bringing your relative to the hospital for mental health services?

R: As for the benefits, I think he is able to release stress and he gets better but he stops taking the medicine and the condition then comes back.

1. How has the mental illness of your relative affected you? Please explain

R: when I was in Kumasi, I was helping with some painting job and I get my own money but now I have to call people and family members who sometimes do not pick because they know you are going to ask for money.

1. How do you cope with these challenges or stresses you experience? Please explain

R: Everything is God so I believe God is the one taking care of me.

1. What do you think should be done to address those factors hindering the utilization of mental health services in this municipality?

R: Ok, I think the government should help, we have to pay for medicines and others. I think the health insurance should cover at least half of the drugs.

**006--**

1. Can you please tell me your age? 58

2. What gender are you? Female

3. What is your ethnicity? Frafra

4. What is your marital status? Divorced

5. How many children do you have? 4

6. Are you educated? To What level, please? Form 4

7. What work do you do? Drug Peddler

8. What is your place of residence? Zuarungu.

9. What is your religion? Christian

10. How are you related to the patient? Mother

1. Before your relative got mentally ill, what was your thinking regarding mental illness?

R: I always thought it was so far from me, normally I though it was those who smoke or are cursed maybe if you go and disrespect elderly people then you can develop mental condition

1. What about your relative made you think he/she was mentally ill?

R: She was fine until when she was 8 years that she had an attack then we sent her to the hospital and we all thought she was fine until she went to secondary school and started talking some unusual things saying she can see dead people or hear voices. This was very strange to all of us

1. How do people in your community think about mental illness?

R: As for my community people, they will insult you and call you mad. If you have an argument with someone, they will say its because you are mad. Others even accuse my daughter of witchcraft. They say they are giving her witchcraft and she is resisting that is why the condition is affecting her

1. What do you think are the causes of your relative’s mental illness?

R: I think maybe some people might have invoke it on her. I think it is spiritual. Because I just cannot see what will cause such a condition.

1. Where did you visit first for the diagnosis? Why?

R:the hospital, because of the seizure, but when the behavior problem started I took her to the church because I thought it was a spiritual problem

1. Where did you visit first for treatment? Why?

R: As for the church, they only prayed for her but in the hospital, they started her on medications.

1. What factors discourage you from bringing your relative to the hospital for mental health service

R: Getting money to bring her to the hospital and also purchasing of the medications has been a challenge for me. Apart from that I don’t have any other challenges. Another challenge is the stigma, because of the accusation of witchcraft and the beliefs about mental illness in my community, I sometimes feel like staying home and keeping my daughter from outsiders.

1. What factors that encouraged you to seek mental health care from Hospital?

R: ooh, when we started coming to the hospital, especially the psychiatric hospital, she has been very fine with no problems again. So I don’t want the problem to come back again.

1. What benefits have you derive from bringing your relative to the hospital for mental health services?

R: Yh, a lot of benefits like I mentioned, she is now working which makes me very happy.

1. How has the mental illness of your relative affected you? Please explain

R: A lot of problems, I am unable to do my work, it has exhausted all my capital. I used to be bigger than this but see, I have even lost weight. It hasn’t been easy for me at all.

1. How do you cope with these challenges or stresses you experience? Please explain.

R: I trust God for his grace to carry me through. I am always praying and believing God.

1. What do you think should be done to address those factors hindering the utilization of mental health services in this municipality?

R: I think a lot of education need to go on so that people will understand mental illness to stop the way they treat people with the condition.

**007**

Can you please tell me your age? 26

2. What gender are you? female

3. What is your ethnicity? frafra

4. What is your marital status? married

5. How many children do you have? nil

6. Are you educated? To What level please?shs

7. What work do you do? Unemployed / apprentice

8. What is your place of residence? Zuarungu

9. What is your religion? Christian

10. How are you related to the patient? husband

a) Before your relative got mentally ill, what was your thinking regarding mental illness?

R: I think it is high fever or malaria, I think that sometimes when you are thinking too much or quarrelling too much with people you can get that problem

b) What about your relative made you think he/she was mentally ill?

R:He started behaving very bad, he even sacked me from the house. He say I have slept with my brother in-laws and that I want to kill him and saying I am something disturbing him so he sacked me.

c) How do people in your community think about mental illness?

R: its true, when your relative has mental illness, they say you the wife you are making him think too much and giving him problems that is why he develop the condition.

d) What do you think are the causes of your relative’s mental illness?

R:I think it is a spiritual thing because it has never happened to him. Since I married him, this is the first time so I think it is maybe a spiritual thing

a) Where did you visit first for the diagnosis? Why?

R:Church, I told my pastor that my husband is behaving different and he said I we should pray first. So I took him to church and they prayed for him

b) Where did you visit first for treatment? Why?

R: it was the hospital that they said he has a mental problem and they started giving him medicine.

c) What factors discourage you from bringing your relative to the hospital for mental health service

R: yh, like he doesn’t want to come to the hospital, he say there is nothing wrong with him. i even told him that as for money problem, he shouldn’t worry because the family people will help him

d) What factors that encouraged you to seek mental health care from Hospital?

R:the way he was, he like talking a lot and attacking me so I wanted him to get well.

e) What benefits have you derive from bringing your relative to the hospital for mental health services?

R: Because of the sickness, he will not sleep and cannot sit at one place but seeing him now he can sit down and sleep well. He hasn’t sacked me again

a) How has the mental illness of your relative affected you? Please explain

R: I don’t have peace, anything you say he think its not good all because of the illness. Also, the people in our community are accusing me and talking bad things about me.

b) How do you cope with these challenges or stresses you experience? Please explain.

R: I am now in prayers; I am talking to the pastors to support me in prayers.

1. What do you think should be done to address those factors hindering the utilization of mental health services in this municipality?

R: The hospital should educate people on mental illness and advise them to understand the condition

**008**

Can you please tell me your age? 57

2. What gender are you? male

3. What is your ethnicity? TALENSI

4. What is your marital status? MARRIED

5. How many children do you have? 3

6. Are you educated? To What level please? TERTIARY

7. What work do you do? PASTOR

8. What is your place of residence? TONGO

9. What is your religion? Christian

10. How are you related to the patient? HUSBAND

a) Before your relative got mentally ill, what was your thinking regarding mental illness?

R: Its not the best at all. I thought it was due to some curse or attacks

b) What about your relative made you think he/she was mentally ill?

R: she started complaining, always accusing you. I did not understand until one day she accused of being interested in other women and that I wasn’t concerned. She got glued to her phone throughout the night and will not sleep. She started complaining about messages on what sup and Facebook and that people were plotting against her on Facebook and that they were discussing her on radio. All these occurred shortly after she gave birth.

c) How do people in your community think about mental illness?

R: some people see it as a curse, maybe you have offended someone and they have placed a curse on you

d) What do you think are the causes of your relative’s mental illness?

R:I think its from stress from giving birth. She started complaining of so much work and wanted her mother to come and support but the mother wasn’t coming

a) Where did you visit first for the diagnosis? Why?

R: Clinic—Maturity is one thing and with my age, I need to know these things, so I needed to pray but the hospital was first

b) Where did you visit first for treatment? Why?

R: hospital, so she could get appropriate medications

c) What factors discourage you from bringing your relative to the hospital for mental health service

R: She didn’t like taking the medications, that was the main challenge. If she had taken the medicine earlier, maybe she would have been fine earlier. You have to shout and encourage her severally before she will take the medicine.

d) What factors that encouraged you to seek mental health care from Hospital?

R: number one, because of what people will say, and also, aside from the prayers I knew that she needed treatment. I also have some understanding about mental illness so I didn’t see it as a challenge to bring her.

The attitude of the staff at the hospital has also been a great motivation.

e) What benefits have you derive from bringing your relative to the hospital for mental health services?

R: When she was discharged, apart from some few days she wasn’t in agreement to take the medicine, she has been well and stable since.

a) How has the mental illness of your relative affected you? Please explain

R: You marry a young lady after your wife passed away and she falls sick, it was very difficult. I will be walking but my mind will not be there. Even till now I still struggle. I have body pains, poor sleep and difficulties concentrating.

b) How do you cope with these challenges or stresses you experience? Please explain.

R: I am seeing it to be normal, though the stress is there. The church has been very supportive too and this has kept me going

1. What do you think should be done to address those factors hindering the utilization of mental health services in this municipality?

R: the attitude of the staff and the way they treat patients should be increased and this will help encourage people to use the service.

**009**

Can you please tell me your age? 43

2. What gender are you? female

3. What is your ethnicity? talensi

4. What is your marital status? widow

5. How many children do you have? 2

6. Are you educated? To What level please? primary

7. What work do you do? trader

8. What is your place of residence? Tongo

9. What is your religion? Christian

10. How are you related to the patient? mother

a) Before your relative got mentally ill, what was your thinking regarding mental illness?9i

R: I think that it is hereditary, and if one is not patient, you can stress yourself to create such a problem for you.

b) What about your relative made you think he/she was mentally ill?

R: he never speaks back at me but my son came to me and accused me of certain things that were untrue. He spoke about things that did not make sense. So, I thought he could be having a mental illness.

c) How do people in your community think about mental illness?

R: They think mental health might be as a result of curses or when you do something wrong against someone, the person may invoke spiritual forces against you but I don’t believe in this though.

d) What do you think are the causes of your relative’s mental illness?

R: what I think is that he is somebody who doesn’t listen to advice so I forgot about him. I think he over thinks, and involves himself in all sorts of deliverance and prophecies. If your faith is not strong whiles you engage is spiritual warfare, other forces might attack you. I think that might have been the cause of this problem

a) Where did you visit first for the diagnosis? Why?

R: Hospital, he left home and went to Techiman. We got the police to assist us to send him to the hospital where they said he might have a mental illness and subsequently gave him some injections but he didn’t sleep nor calm down

b) Where did you visit first for treatment? Why?

R: Hospital

c) What factors discourage you from bringing your relative to the hospital for mental health service.

R: well, he refused coming claiming that there was nothing wrong him and also, I didn’t have have money to afford the treatment. These were my major challenges. Another one was the influence from family where they even pushed me to leave him at a church for prayers for a period of one week even against my will.

d) What factors that encouraged you to seek mental health care from Hospital?

R: I think the way your staff and doctors listened to us made encouraged me to bring him subsequently. The staff also motivated us to believe that our situation wasn’t the worst and that there was solution.

e) What benefits have you derive from bringing your relative to the hospital for mental health services?

R: he used to accuse me of wanting to kill him but now he is no more like that. He also used to say there was nothing wrong with him but now he has admitted that he is not well. I think it’s a good improvement

a) How has the mental illness of your relative affected you? Please explain

R: a lot of effects has come upon me, I have had to spend so much money in tracing him when he leaves home and travel to other places. It has also affected my physical health, I couldn’t sleep, couldn’t eat and worry excessively. I have now developed stomach ulcer as a result of the starvation I went through during following him around.

b) How do you cope with these challenges or stresses you experience? Please explain.

R: I have taken it as a temptation that I’m going through. I trust God that since he gave him to me, he will surely heal him for me.

1. What do you think should be done to address those factors hindering the utilization of mental health services in this municipality?

R: The way you people have started I think its good, your patience for the patients is a good thing and I will encourage you to keep it up.

**010**

Can you please tell me your age? 59

2. What gender are you? female

3. What is your ethnicity? kusaasi

4. What is your marital status? divorced

5. How many children do you have? 2

6. Are you educated? To What level please? Form 4

7. What work do you do? seamstress

8. What is your place of residence? Tiili

9. What is your religion? christian

10. How are you related to the patient? mother

a) Before your relative got mentally ill, what was your thinking regarding mental illness?

R: when we go for prayers, they say mental illnesses are due to spiritual attacks. Apart from that I don’t know

b) What about your relative made you think he/she was mentally ill?

R: you will see her laughing alone, picking peoples things and entering peoples rooms. She sometimes also talks to herself.

c) How do people in your community think about mental illness?

R: as for mental illness, it can affect everybody, it may be spiritual or as a result smoking hard drugs. They also believe that sometimes, if there are spiritual calling and one refuses, they may start behaving like mental illness

d) What do you think are the causes of your relative’s mental illness?

R: I don’t know, but when we go for prayers they say its spiritual

a) Where did you visit first for the diagnosis? Why?

R: she was first brought to the spiritual healer in her village because the symptoms were unusual and we heard that it was spiritual

b) Where did you visit first for treatment? Why?

R: Traditional healer: the father believed that it was spiritual hence the traditional healer was the best place for treatment

c) What factors discourage you from bringing your relative to the hospital for mental health service

R: I am not discouraged but she will not accept that she is unwell, and refuses to accept the treatment.

d) What factors that encouraged you to seek mental health care from Hospital?

R: I know if she takes her medications, she will be fine, so that encourages me to keep bringing her for treatment

e) What benefits have you derive from bringing your relative to the hospital for mental health services?

R: she gets well on treatment; the only problem is when she stops taking the medications

a) How has the mental illness of your relative affected you? Please explain

R: I have lost so much money as a result of the condition. I also think so much causing me sleepless nights because by now she would have been employed and living her own life somewhere

b) How do you cope with these challenges or stresses you experience? Please explain.

R: Usually when I am in the market and these thoughts become strong, I resort to listening to the radio to console myself. This makes me forget of all the problems of my daughter at least for a short while.

1. What do you think should be done to address those factors hindering the utilization of mental health services in this municipality?

R: If there could be financial support for the patients, that will help since its very expensive seeking help.

**011**

Can you please tell me your age? 33

2. What gender are you? male

3. What is your ethnicity? Ewe

4. What is your marital status? single

5. How many children do you have? nil

6. Are you educated? To What level please? tertiary

7. What work do you do? Agribussiness

8. What is your place of residence? Zuarungu

9. What is your religion? None

10. How are you related to the patient? Brother

a) Before your relative got mentally ill, what was your thinking regarding mental illness?

R: honestly speaking, I have been ignorant about mental illness, we thought it is spiritual or something

b) What about your relative made you think he/she was mentally ill?

R: She beat up our old mother which made me feel this was very unusual. She also claims she has been ordered to go and deliver somebody who is ill, rather shocking

c) How do people in your community think about mental illness?

R:We believed it is caused bey marijuana and also if people are involved in ritual monies, they may develop such conditions.

d) What do you think are the causes of your relative’s mental illness?

R: Self-denial is one of the things I believe might have caused her problem, she refuses to take her medicines

a) Where did you visit first for the diagnosis? Why?

R: Regional Hospital. because she had attempted suicide claiming she was the black sheep of the family

b) Where did you visit first for treatment? Why?

R: Regional Hospital, since she was being managed for the suicide attempt so they ended up treating her

c) What factors discourage you from bringing your relative to the hospital for mental health service

R: I have been ignorant about mental health and even more not knowing there is a place for treatment such as this. Also the attitude of nurses, the way they look at us when we mention that we are coming to the psychiatric hospital.

d) What factors that encouraged you to seek mental health care from Hospital?

R:This is definitive care, we have been in and out of various facilities hoping for results so when we were told about psychiatric hospital, we were motivated to bring her for treatment. This treatment also looks more humane than taking her to places for chaining and physical abuses

e) What benefits have you derive from bringing your relative to the hospital for mental health services?

R: well, she gets better when she complies with her treatment. So we think that if she is able too take her treatment, she will be fine

a) How has the mental illness of your relative affected you? Please explain

R: I have had to leave my work and take her to the hospital for treatment. Its been quiet time consuming and stressing for.

b) How do you cope with these challenges or stresses you experience? Please explain.

R: Well, its fine we are encouraged by our quest for see her recover

1. What do you think should be done to address those factors hindering the utilization of mental health services in this municipality?

The way staff view mental illness I think it’s bad such attitudes need to change. Also, you will need a sign board to direct and communicate with people to be able to know where

**012**

Can you please tell me your age? 27

2. What gender are you? Male

3. What is your ethnicity? Talensi

4. What is your marital status? Single

5. How many children do you have? Nil

6. Are you educated? To What level please? SHS

7. What work do you do? TILER

8. What is your place of residence? Bolgatanga

9. What is your religion? Christian

10. How are you related to the patient? Brother

a) Before your relative got mentally ill, what was your thinking regarding mental illness?

R: well, when someone is doing things, he is not supposed to do or saying somethings he is not supposed to say, we can say he has a mental illness. Also, it may be caused by sometimes the things you take like smoking or drinking, or maybe nature like in the family is the cause of mental illness.

b) What about your relative made you think he/she was mentally ill?

R: like if you say something or ask a question, he will say a different thing, he will remove his clothes or be talking to himself as if he is conversing with somebody

c) How do people in your community think about mental illness?

R: they accuse people with mental illness to be drug addicted, or maybe doing something that doesn’t go with him that’s why he has this condition

d) What do you think are the causes of your relative’s mental illness?

R: one if he was a drug person, I would say it’s that one but now, its just hard. I can’t really say what the cause is

a) Where did you visit first for the diagnosis? Why?

R: The regional hospital, they actually said he has a mental illness. We thought that’s the place where he will get help.

b) Where did you visit first for treatment? Why?

R: The regional hospital, since they said he it was a mental illness, we thought he should continue the treatment there

c) What factors discourage you from bringing your relative to the hospital for mental health service

R: He refuses to take his medicine, that is the main challenge. As for the other things about money, we don’t think that is a problem. If only he will take the medicine and get well, we will be very happy

d) What factors that encouraged you to seek mental health care from Hospital?

R: I want him to get well, we even thought it had gone for good only for it to come up after 2 years.

e) What benefits have you derive from bringing your relative to the hospital for mental health services?

R: He is far better now. Better that we brought him. When he is well, he is able to do his work but when the condition come up, he is unable to do anything.

a) How has the mental illness of your relative affected you? Please explain

R: So much, now there is work but I cannot do it. I am supposed to be at the site but im here. Also, because of taking care of him, I cannot go and make money

b) How do you cope with these challenges or stresses you experience? Please explain.

R: I am hopeful that he will get well this is what keeps me going

1. What do you think should be done to address those factors hindering the utilization of mental health services in this municipality?

R: I wish there was such a service where people could go to specifically for mental healthcare where there are experts to treat them but before now, there was no such places and made us roam a lot. These services should be available so that we can easily use them.

**013**

1. Can you please tell me your age? 56

2. What gender are you? female

3. What is your ethnicity? Frafra

4. What is your marital status? widow

5. How many children do you have? 4

6. Are you educated? To What level, please? nil

7. What work do you do? trader

8. What is your place of residence? Bolgatanga

9. What is your religion? Christian

10. How are you related to the patient? Mother

1. Before your relative got mentally ill, what was your thinking regarding mental illness?

R: Well, I don’t know anything about mental illness. I only see people walking around and we call them mad but what causes them or what they do, I cannot tell until my son’s own condition began.

1. What about your relative made you think he/she was mentally ill?

R: When it started, he was Refusing meals, became physically aggressive and could not sleep and we had to bring a nurse to inject him. he got well and it came again.

1. How do people in your community think about mental illness?

R: well there are people walking around some almost naked, talking anyhow. They say, its due to marijuana and some alcohol. That is all I have seen and heard.

d) What do you think are the causes of your relative’s mental illness?

R: he has been working with a woman who sells “soobolo” laced with “weed” so I suspect he might have drunk some of the drink which might have caused his condition.

1. Where did you visit first for the diagnosis? Why?

The first time, we called a nurse who came to the house to give him an injection, she said he had a mental illness. His brother is a nurse so he said we should call the nurse to come.

b) Where did you visit first for treatment? Why?

The nurse again was the first to treat him and we continued with the treatment because he got better after the first treatment.

1. What factors discourage you from bringing your relative to the hospital for mental health service.

R: Financial challenges have been my main problem, anytime we have to bring him, we will need to get a vehicle, pay for the transportation and also pay for the medications which have become a challenge for me. Also, he refuses to take the medicine when he is well and very difficult to get him to come for his reviews.

1. What factors that encouraged you to seek mental health care from Hospital?

R: We heard on the radio that there is a place in Bolgatanga where they only treat mental illness and that if we bring him to the hospital, he will be well. That’s what motivated me to bring him to the hospital.

1. What benefits have you derive from bringing your relative to the hospital for mental health services?

R: A lot of benefits, now, he is getting better and instead of attacking me like previously, he receives me well now and even requests for food. I am able to communicate with him and the communications is meaningful unlike before. I am really excited now about his improvement.

a) How has the mental illness of your relative affected you? Please explain

R: I have lost weight, unable to eat and have been crying a lot especially in the night. I have also lost all my business capital in the treatment.

1. How do you cope with these challenges or stresses you experience? Please explain

R: I have been praying to God to heal my son completely. I believe all shall be well. With this faith, I am able to move on.

1. What do you think should be done to address those factors hindering the utilization of mental health services in this municipality?

R: Public education on mental illness is very important. I suggest radio announcement about where to send people with mental illnesses for care. This was way, many people will know where to send their mentally ill pattients and not keep people with mental illness in the house.

**014**

1. Can you please tell me your age? 32

2. What gender are you? male

3. What is your ethnicity? frafra

4. What is your marital status? married

5. How many children do you have? 1

6. Are you educated? To What level, please? tertiary

7. What work do you do? Nurse

8. What is your place of residence? Zaare

9. What is your religion? Christian

10. How are you related to the patient? Brother

1. Before your relative got mentally ill, what was your thinking regarding mental illness?

R: I think mental illnesses can be caused by genetic factors, and can also be caused by drugs of abuse. People with mental illnesses may not be able to keep up with their daily activities, neglect their personal hygiene and also doing what they normally will not do.

1. What about your relative made you think he/she was mentally ill?

R: he was refusing food, refusal to talk and behaving in an unusual way for about 14 days. That was when he was sent to the hospital

1. How do people in your community think about mental illness?

R: the people see it to be spiritual or under the influence of hard drugs. Most people are not enlightened about mental illness.

1. What do you think are the causes of your relative’s mental illness?

R; I think his case can be due to alcohol and smoking. Even though I have never seen him drink nor smoke, investigations revealed that he does all these things.

1. Where did you visit first for the diagnosis? Why?

R: we first sent him to the regional hospital for diagnosis. This is because, he had attempted suicide before so we had to send him to the regional hospital when we suspected mental illness

1. Where did you visit first for treatment? Why?

R: The regional hospital was the place where they initiated treatment, like I said because of his previous suicidal attempt, we felt the hospital was the best place to treat him

1. What factors discourage you from bringing your relative to the hospital for mental health service.

R: I think the difficulty is the lack of support and I am the only one runming having to leave my job and come here to take care of him. Also, financial difficulties are the challenges I go through

d) What factors that encouraged you to seek mental health care from Hospital?

I didn’t know about the presence of a specialist in the region, it was a friend who told me about it hence I felt the need to bring him for care. Also, as a human being, I wish he gets well and able to live his normal life. These are the factors that motivated me to bring him here

1. What benefits have you derive from bringing your relative to the hospital for mental health services?

R: There has been a lot of benefits. Initially I lost hope, but seeing him recover am most grateful

1. How has the mental illness of your relative affected you? Please explain

R: it has affected me financially, also as a married person, I neglect my family to spend a lot of time taking care of my brother. There are times I have to use the housekeeping money to take care of the hospital bills and if not for my understanding wife, it would have been difficult.

1. How do you cope with these challenges or stresses you experience? Please explain

R: As a Christian I believe everything happens for a reason, so I know things will get better.

1. What do you think should be done to address those factors hindering the utilization of mental health services in this municipality?

R: for now, I think that management of such a system is difficult. I think the team is doing well and I will encourage that this facility is expanded so that there are more of such facilities in the region to treat people with these conditions. Another thing is also the financial support, if there is any financial support, I think it will help encourage and motivate many people to come for treatment.

**015**

1. Can you please tell me your age? 39

2. What gender are you? male

3. What is your ethnicity? frafra

4. What is your marital status? married

5. How many children do you have?

6. Are you educated? To What level, please? jhs

7. What work do you do? security

8. What is your place of residence? via

9. What is your religion? traditionalist

10. How are you related to the patient? Brother

1. Before your relative got mentally ill, what was your thinking regarding mental illness?

R: the way people talk; their behavior and their actions can point to mental illness. Sometimes, when what you are saying is not bad but can take it as bad. They can say you want to kill them or do something bad to them meanwhile you don’t plan anything like that. As for their treatment, I think both the traditionalist, the church and the hospital all can help.

1. What about your relative made you think he/she was mentally ill?

R: sometimes he will call me and the way he will talk about things that are not normal. He will say things that are not related to the conversation, you will know that he is not himself. he is unable to sleep even day and night.

1. How do people in your community think about mental illness?

R: At times they say it is as a result of smoking a curse or other times the cause is not known. The people in via will still come closer to you even though you have mental illness

1. What do you think are the causes of your relative’s mental illness?

R: I can say he has been thinking too much

a) Where did you visit first for the diagnosis? Why?

R: we first took him to the hospital, we believe that if anything happens, we have to first go to the hospital if the needs water or blood, it’s the hospital that can help.

1. Where did you visit first for treatment? Why?

R: because he could not sleep, it’s that hospital that can give him medicine to sleep.

1. What factors discourage you from bringing your relative to the hospital for mental health service

R: money is always the problem but we still try.

d) What factors that encouraged you to seek mental health care from Hospital?

R: As for human beings, health first so the hospital is the place where he can get the help he needs. We want him to be healthy so we brought him

1. What benefits have you derive from bringing your relative to the hospital for mental health services?
2. A lot. Now all he is not behaving abnormally again; he is well and we can relate well.
3. How has the mental illness of your relative affected you? Please explain

R: I can’t go to work, I can’t leave him alone and go back to my house to take care of my family.

1. How do you cope with these challenges or stresses you experience? Please explain

R: As a human being I know problem will come but sometimes, it will settle so I keep on helping

1. What do you think should be done to address those factors hindering the utilization of mental health services in this municipality?

R: I think there is a need for expansion of the mental health service so that more people can be admitted if they need the service.

Also, medicine is a problem and the patient has to buy for themselves. People too may not have a means of transportation to bring their relatives for care. I think these problems need to be fixed.

CARE PROVIDER 1

1. Can you please tell me your age? 35

2. What is your gender? Female

3. Where do you residence please? yikene

4. Are you married? Married

5. What is your highest level of education? Tertiary

5. How long have you been working in Mental health? 2 years

6. What is your job title? Nursing Officer

7. Which religion do you belong to? Christianity

1. What are the major Mental services provided by this facility?

R: We provide both opd services and inpatient services, psychological services, Children also come so we see children with mental health challenges including addiction.

1. Is the delivery of Mental health services by this facility of good quality? If yes/no, please explain.

R: I will say very good, because our patients recover well, the patients tell us how good our services are.

1. Do people from this community bring their mentally ill relatives for Mental health services?

R; Yes, they do,

1. What factors have you identified as facilitators of the utilization of mental health service in the municipality?

R: The presence of a specialist in the municipality has increased the number of patients who attend the mental health service since previously they needed to travel down south to meet a specialist psychiatrist but now so close them , availability of drugs which are not readily available for the patients makes the clients feel satisfied, and also when they come, the care and attention they receive encourages them to come.

1. What factors have you identified as barriers to the utilization of mental health service in the municipality?

R: the challenge is mainly about the payment; most are unable to afford the cost of care and cost of medications.

Also in a way, some believe that the condition is spiritual hence resort to the spiritual healers but end up still coming to us because they don’t get well at the faith healers. some also believe that some of their conditions get worst with injections hence stops them from coming,

Staffing challenges also impair the use of the service because they end up delaying or spending too much time here when they attend the clinic.

Also, the absence of any form of health insurance is a challenge which hinders people from utilizing the service

1. What are your recommendations for improving mental health service utilization at this municipality?

R: Education should be intensified since most of our people are ignorant about mental health hence resort to faith healers.

There is the need to step up the staffing levels to raise the level of services provided in the municipality since this leads to unnecessary delays at the facilities with clients complaining bitterly.

CARE PROVIDER 2

1. Can you please tell me your age? 33

2. What is your gender? female

3. Where do you reside please? Yorogo

4. Are you married? married

5. What is your highest level of education? tertiary

5. How long have you been working in Mental health? 4

6. What is your job title? Staff Nurse

7. Which religion do you belong to? Christianity

1. What are the major Mental services provided by this facility?

R: we do detox, other forms of mental health cases including child and adolescent cases, old age psychiatry and a lot more.

1. Is the delivery of Mental health services by this facility of good quality? If yes/no, please explain.

R: if I am to grade from 0-10, I will say 9. Because our patients recover well and go back to their normal life so I can say so far so good, we are doing well.

1. Do people from this community bring their mentally ill relatives for Mental health services?

R: Yes please

1. What factors have you identified as facilitators of the utilization of mental health service in the municipality?

R: people give good comments about our services to them so they are encouraged to come, Also the rapport we establish with them also encourages them to keep coming because, without good rapport, they will be discouraged to come. They also take cues from their community members who have had benefits from using this service. Another point I think has helped is that we were recently trained by the mental health authority of Ghana on quality rights in mental health and this has transformed our work so people feel safer with the service than before.

1. What factors have you identified as barriers to the utilization of mental health services in the municipality?

R: Sometimes, financial challenges are their main problems because the people here are poor and find it difficult to pay.

Also, because mental health is cash and carries without any form of health insurance, they are unable to pay.

I: What are your recommendations for improving mental health service utilization at this municipality?

R: if the government could absorb the medication cost or cover the service with a health insurance package, it would encourage people to keep coming.

MENTAL HEALTH SERVICE ADMINISTRATOR 1

1. Can you please tell me your age? 40

2. What is your gender? Male

3. Where do you reside please? Yarigabisi in Bolga

4. Are you married? yes

5. What is your highest level of education? Tertiary

5. How long have you been working in Mental health? Over 15 years

6. What is your job title? I am a Psychiatric nurse and also the Municipal mental health coordinator.

7. Which religion do you belong to? I am a Christian.

I: What do you think about the Mental health situation of this municipality?

R: Well, I think I will grade it as a little above average. We are not top notch yet but we are above the average too. At least we are one of the few regions with a psychiatrist and also a new facility dedicated to mental health care. So I think we are gradually getting there.

I: What are the major Mental health services provided by the health facilities in this municipality.

R: Ooh well, we provide mainly opd services, we don’t have a psychologist now so we don’t do a lot of psychotherapies. Now with the dedicated psychiatric facility for there is a psychiatrist, we are able to admit for specialist care. I think addiction management is also going on in the municipality too.

I: What are the factors that hinder the utilization of mental health services by people in this municipality.

R: Well, you know, in this part of the country, the poverty is too much hence people are unable to afford their medications. When one needs to be admitted, they need to pay. This is very difficult for most people hence they are unable to afford the service.

I: Are there anymore factors you will like to add?

R: hmmm, I think their beliefs too affect the use, some believe that mental illness is caused by some spiritual forces hence they prefer going to the Churches and traditional healers for treatment. They only come to us when they fail.

I: is that all?

R: that’s all I can say for now.

I: What are the factors that facilitate the utilization of mental health services by people in this municipality.

R: Now that we have a specialist in the region, people seem to be encouraged to come for care. Previously, it was the nurses alone you could find but now, there is a specialist and I think it has helped.

I: is that all you can think about?

R: In my clinic, the clients tell me they are more comfortable using our service now than before. Our client numbers have increased over the last year. I think the increase is because of the quality rights training and implementation.

I: What will you recommend for the improvement of mental health service utilization in this municipality?

R: I think we need more education for the people to understand mental health, to reduce the stigma and also to stop believing in the faith healers.

I: what else do you think should be done?

R: hmm, the issue of mental illness not being covered by NHIS is something very bad and needs to be looked at.

I: Is there anything more you want add?

Nothing more for now.

I: thank you so much for your time.

MENTAL HEALTH ADMINISTRATOR 2

Can you please tell me your age? I am 43 years old

2. What is your gender? Male

3. Where do you residence please? Around the SSNIT office

4. Are you married? yes

5. What is your highest level of education? Postgraduate Degree, Masters I mean

5. How long have you been working in Mental health? Oooh, about 20 years… since 2003

6. What is your job title? I am the Regional Mental Health coordinator but also a mental health nurse.

7. Which religion do you belong to? Christian

I: What do you think about the Mental health situation of this municipality?

R: I think it’s still underdeveloped. I mean most people still prefer to use alternative treatments instead of MHS for their mental health problems. We still don’t have a psychologist in the region and hence none in the municipality. We can however admit to a dedicated facility though.

I: What are the major Mental health services provided by the health facilities in this municipality.

R: Mainly OPD services since there is only one psychiatrist. Admissions are done at the specialist hospital. That is where addiction management, Child and adolescent mental health services are rendered too. Also, I think geriatric psychiatric services are also rendered there.

I: What are the factors that hinder the utilization of mental health services by people in this municipality.

R: I believe that the main challenge I have seen is ignorance about mental health in general. It is the reason why even though there is treatment and sometimes free, people still prefer to spend huge sums of money with alterntive care. Also, the non inclusion of mental health into the health insurance scheme. These people are the vulnerable in society but they have been excluded.

I: Is that all you can say?

R: Well these are the main points I have found from my 20 years’ experience.

I: What are the factors that facilitate the utilization of mental health services by people in this municipality.

R: Thanks to the passage of the mental health act, mental health services have been made free even though sometimes there are no medications and hence people have to buy but it has encouraged people to use the service better than before. Another one I think about is the presence of a dedicated facility where people can be admitted. Even though people may say that will breed stigma, I think it is also the best since the staff there will understand the clients better and treat them better.

I: any more please?

R: That’s all I can say now.

I: What will you recommend for the improvement of mental health service utilization in this municipality?

R: There are several things I think should be done but immediately, I think more education should go into creating awareness of the communities about mental health and illness to reduce stigma and encourage people to use the service. Also, I think as a matter of urgency, mental health service should be included into the NHIS. This will take away the financial burden on the families and hence they bring their ill relatives to the MHS.
