# Supplementary material for: Exploring the burden on family caregivers in providing care for their mentally ill relatives in the Upper East Region of Ghana
Source: PLOS Glob Public Health. 2024 Apr 16;4(4):e0003075. doi: 10.1371/journal.pgph.0003075 (PMC11020355; doi:10.1371/journal.pgph.0003075)
Supplement: Attachment — Submitted filename: Point by point response 02_01_2024.docx [file pgph.0003075.s002.docx]

Editorial comments

We noticed that you used "unpublished" in the manuscript. We do not allow these references, as the PLOS data access policy requires that all data be either published with the manuscript or made available in a publicly accessible database. Please amend the supplementary material to include the referenced data or remove the references.

**Response
After revising the manuscript, we do not have any reference as unpublished**

2. We have noticed that you have a list of Supporting Information legends in your manuscript. However, there are no corresponding files uploaded to the submission. Please upload them as separate files with the item type 'Supporting Information'.

**Response**

**This has been specified**

3. In the online submission form, you indicated that "Data for the study can be found in the study as excerpts. Full data will be available from the corresponding author upon reasonable request". All PLOS journals now require all data underlying the findings described in their manuscript to be freely available to other researchers, either 1. In a public repository, 2. Within the manuscript itself, or 3. Uploaded as supplementary information.

**Response**

**We have uploaded the data as supplementary information**

**Dear Editor**

**We would like to thank you sincerely for the insightful reviewer comments on our manuscript. We find the comments very useful and have responded to them to the best of our knowledge. We acknowledge that the comments have no doubt helped improve the quality of our manuscript. We herein respond to detail how we have addressed the comments of the reviewers point-by-point. For easy identification, the reviewers’ comments have been repeated while the Authors’ responses appear in bold text.**

Introduction is very well structured and describes the scientific field satisfactory. In this part the authors refer to studies from other countries. Why were these studies selected? Do they have some socioeconomic similarities to Ghana? Or were there other selection strategies that resulted in these references.

**Response**

**Authors in this study sought to review literature from the global level (international), down to the local context (Ghana). We therefore sought literature that had information in the study area from across the globe using search engines such as Google Scholar, PubMed, Scopus among others.**

In describing the aim of the study, the authors state that similar studies have been conducted in southern Ghana, and not in the northern part. Nevertheless, they state that the aim of the project was to study burden of relatives in the whole of Ghana. This should be clarified.

**Response**

**This has been clarified.**

The sentence: P10 To ensure credibility, space triangulation, thus, the collection of data from different sites was done seems incomplete to me. Please clarify the role of «space triangulation» in this sentence.

**Response**

**The statement has been completed and the role of space triangulation has been explained.**

P10: Participants were mainly females (53.3%) with a mean age of 42.74 ±14.2 and most of them were mothers to mentally ill patients as shown in Table
I would say «mainly» is imprecise, because you recruited almost similar numbers of male and female participants. Suggested rephrasing: Eight participants were female of which seven were mothers, and seven were male of which four were brothers. Mean age was 43 years (SD 14).

**Response**

**We have clarified the sentence**

Results
**Table 2 Thematic analysis.**
The authors should clarify whether themes and sub-themes were defined prior to data collection or as part of the analysis. If defined prior to, the authors should not state in Discussion that «Three main themes emerged including social burden, physical burden, and psychological burden».

**Response**

**We have explicitly explained in the analysis section that an inductive approach was used in arriving at themes and subthemes. Thank you**
I’m not quite sure that the label «physical burden» is relevant for the somatic symptoms, because the symptoms, as presented in quotes, are clearly related to emotional/psychological distress.

**RESPONSE**

**Thank you for the attention to this, we have replaced the quotes with a lot more specific quotes.**

Phrase: P14 «in tandem» please re-phrase if you mean that the finding is in accordance with, similar to or concordant with

**Response**

**This has been rephrased.**

Reviewer #2: Abstract

1. "Care for the mentally ill is saddled with several challenges financial difficulties, stigma, and psychosocial challenges among others."
Consider rephrasing for clarity: "Caring for the mentally ill involves numerous challenges, including financial difficulties, stigma, and psychosocial issues, among others."

**Response**

**We have rephrased the sentences as suggested**

2. "It is also imperative to include mental health services in the national health insurance scheme to alleviate the financial burden of family caregivers." Consider rephrasing for emphasis: "Additionally, it is imperative to integrate mental health services into the national health insurance scheme to alleviate the financial burden on family caregivers."

**Response**

**We have rephrased the sentences as suggested**

Introduction
3. The Introduction section of the paper appears to be inadequately written and does not meet the expected standards for journal submissions. The phrasing and structure could be improved for better clarity and coherence, aligning with the rigorous standards expected in academic publications.
Such as
“Evidence shows that one in every eight people around the world lives with a mental illness.” – The authors have provided only one reference for this claim and that too is a WHO key facts. Citing recent studies based on mental illness would be appreciated.

**Response**

**We have searched for recent studies on this particular statement and replaced the reference with a recent article.**

4. The introduction covers a wide range of topics related to caregiver burden, from global prevalence to specific factors influencing burden in different countries. Consider organizing the information in a more structured manner, perhaps starting with a general overview and then gradually focusing on the specific context of Ghana.

**Response**

**This has been done, the background of the study has been reviewed**

5. Use transitional phrases to guide readers through the logical flow of information. For example, phrases like "Building on this evidence" or "In the context of Ghana" can help connect ideas more smoothly.

**Response**

**This has been done**

6. When discussing caregiver burden in Ghana, try to incorporate specific examples or statistics to illustrate the extent of the issue. This can add depth to the introduction and make it more compelling.

**Response**

**More context has been provided in the discussion. Thank you**

7. Clearly articulate the research gap that this study aims to address. While it's mentioned that studies in northern Ghana are limited, explicitly stating the gap and its significance will provide a stronger rationale for your research.

**RESPONSE**

**This has been done please.**

8. The research question is clear, but you may consider providing a brief rationale for why this question is essential and how its answer contributes to the existing knowledge on caregiver burden in Ghana.
 **Response**

**This has been done**

Methods
9. Please provide a justification for the age criterion (18 years or older) for family caregivers. Why was this age range chosen, and how does it contribute to the study's objectives?
**Response**

**This has been clarified.**

10. In the sentence, "Fifteen in-depth interviews were conducted in all, including four in the Grune language," it might be more helpful to specify if these four interviews were conducted with participants who spoke Grune as their primary language.

**Response**

**We have clarified the statement**

Discussion
11. The integration of previous studies (Azman et al., Addo et al., etc.) is effective in supporting your findings. Consider expanding on the differences or similarities between your study and the cited studies to provide a deeper understanding of the context.
**Response**

**The sentence has been rephrased.**

12. Emphasize the policy implications more explicitly. For example, when discussing financial challenges, clearly state the policy recommendations or changes that could alleviate these challenges, such as the inclusion of mental health services in the National Health Insurance Scheme (NHIS).

**Response**

**We have rephrased the sentence to emphasize the policy implications**

13. Consider including a brief section on potential avenues for future research. What aspects of caregiver burden could be explored further, and what methodologies might be employed?

**Response**

**This has been included. Thank you.**

14. The authors can end the conclusion with a strong call to action, summarizing the key recommendations and emphasizing their importance for the well-being of family caregivers and mentally ill relatives.

**Response**

**The key findings have been highlighted in the conclusion following some recommendations.**
