# Supplementary material for: Exploring the burden on family caregivers in providing care for their mentally ill relatives in the Upper East Region of Ghana
Source: PLOS Glob Public Health. 2024 Apr 16;4(4):e0003075. doi: 10.1371/journal.pgph.0003075 (PMC11020355; doi:10.1371/journal.pgph.0003075)
Supplement: Attachment — Submitted filename: Point by point response 19_02_2024.docx [file pgph.0003075.s003.docx]

**Dear Editor**

**The authors wish to thank you sincerely for in-depth review of our manuscript. Indeed the reviewer comments have no doubt improved the manuscript.**

**The reviewers comments have been repeated while the authors response appear in black bold text.**

We noticed you have some minor occurrence of overlapping text with the following previous publication(s), which needs to be addressed:

- <https://doi.org/10.1111/ppc.12151>

**Response**

**We have revised the sentence to prevent any overlap**

Reviewer #1: This is almost fit for publication. I think new title is more precise, but still, somehow I miss the original.

**Response**

**We believe this title is more clear and concise**

There are a few phrases, misspelling etc that should be corrected, such as:
p21: "In a separate study..." - reconsider phrasing, use "another" instead?

**Response**

**This has been corrected**

p21: they face whiles caring - please correct

**Response**

**This has been corrected**

p21: Researchers have documented -reconsider choice of word, use "showed" instead?

**Response**

**This has been corrected**

p22: therefore creates a a contextual - please correct

**Response**

**This has been corrected**

p27: quote: sometimes do not pick because they know you - is translation correct? alternatively: do not pick up the phone

**Response**

**This has been corrected**
